# Isotretinoin on TikTok: A Qualitative Analysis of Attitudes, Perspectives, and Knowledge Dissemination of User-Generated Content

**DOI:** 10.7759/cureus.80244

**Published:** 2025-03-08

**Authors:** Akua O Asare, Adwoa N Sefa, Leonie Haddad, Sian El-Louise Obeng

**Affiliations:** 1 General Medicine, Guy's, King's and St Thomas' (GKT) School of Medicine, Guy's Campus, King's College London, London, GBR; 2 General Medicine, East Surrey Hospital, Surrey and Sussex Healthcare NHS Trust, Redhill, GBR

**Keywords:** acne vulgaris, clinical dermatology, dermatology trends, healthcare social media, isotretinoin, social media applications, social media perception, tiktok

## Abstract

Background: Acne vulgaris is an inflammatory skin condition that commonly affects adolescents and adults, which correlates well with the main demographic of TikTok (ByteDance Ltd., Beijing, China) users. This study investigates TikTok's role in disseminating knowledge, attitudes, and perspectives regarding isotretinoin among its users.

Methods: This study examined 50 TikTok videos related to isotretinoin. Independent reviewers performed thematic analysis, identifying key themes. Data on views, likes, comments, shares, hashtags, captions, and creator demographics were collected.

Results: Out of 50 videos, 14 had view count data (totaling 607,700 views). All videos had likes, comments, and shares (totaling 950,805 likes, 12,172 comments, and 13,195 shares). Video demographics included 42% of healthcare professionals (18 doctors/dermatologists, two pharmacists, and one dermatology nurse) and 58% of non-professionals (28 patients and one aesthetician), a significant percentage. The most frequently occurring hashtags were #accutane, #isotretinoin, #acne, #roaccutane, and #acnetreatment. The thematic analysis revealed eight themes related to the role of social media in shaping perceptions of the drug. These themes include psychological side effects and impact, personal experiences, treatment process and decision-making, awareness, misconceptions and communication, post-treatment considerations, and outlook. Notably, the discourse is dominated by non-medical content creators who often spread misinformation, particularly regarding mental health risks.

Conclusions: This study highlights TikTok's role in shaping perceptions of isotretinoin, revealing diverse user experiences and the impact of social media on health information. Healthcare professionals must engage with platforms to counteract misinformation and support informed decision-making. Future research should explore ways to improve the quality of health information on TikTok and evaluate its long-term effects on isotretinoin prescription uptake and treatment adherence.

## Introduction

Acne vulgaris, affecting approximately 9% of the global population, primarily impacts individuals aged between 12 and 24 years, the predominant age group of TikTok (ByteDance Ltd., Beijing, China) users [1]. This study investigates the attitudes, perspectives, and knowledge dissemination of isotretinoin among TikTok users, focusing on the platform's effectiveness in sharing information on isotretinoin for acne treatment. The selection of isotretinoin as a focus for the study is justified by its effectiveness in treating acne, a common inflammatory skin condition affecting both adolescents and adults. Its significant role in acne treatment, as well as the fact that guidelines indicate that isotretinoin is typically prescribed in specialist care settings by medically licensed dermatologists for individuals with severe acne that has not responded to other treatments, or in cases where there is significant scarring, a high risk of scarring, or when acne is causing substantial psychological distress, makes it a key subject for investigation.

Isotretinoin is an oral systemic retinoid medication effective in treating acne at a pharmacologic dosage of 0.5 to 1.0 mg/kg per day. Despite its unclear exact mechanism of action, isotretinoin inhibits sebaceous gland function and keratinization, reducing sebaceous gland size and sebum production. It is indicated for severe, resistant, nodular-cystic acne unresponsive to conventional therapies [2,3]. However, there is a notable lack of public knowledge, particularly among teenagers, about isotretinoin's use, side effects, and monitoring requirements. Social media platforms like TikTok have become popular sources of health information, yet a literature review reveals only four articles on PubMed that specifically explore health information dissemination of isotretinoin on TikTok [4-7].

One article reveals that most videos highlight before-and-after improvements in acne severity, with comments primarily discussing side effects and personal experiences. This study suggests that while TikTok videos may shape perceptions and expectations of isotretinoin, they also encourage patient interest in exploring treatment options and facilitate discussions with healthcare providers [4].

Another study highlights the need for dermatologists to engage with trending social media content to better comprehend patient attitudes toward treatments like isotretinoin. Practical approaches, such as hashtag searches to identify popular content, are recommended. This enables dermatologists to anticipate potential misinformation and better address patient concerns and perceptions regarding isotretinoin and acne treatment. Staying informed about social media trends allows dermatologists to offer patient-centered care that aligns with patients' perspectives and needs [5].

A cross-sectional study of popular TikTok videos on isotretinoin revealed issues concerning misinformation, particularly regarding mental health risks. Non-medical content creators largely dominate this discourse, exacerbating the dissemination of misinformation. This detrimentally influences public perceptions and adherence to isotretinoin treatment. Consequently, the study explores the imperative for increased involvement of healthcare professionals in social media spaces to counteract and offer reliable medical guidance [6].

The above sentiment is also echoed by the final article, which discusses the prevalence of misinformation regarding the mental health effects of isotretinoin on TikTok. Despite recent research indicating a lower risk of psychiatric side effects, many TikTok videos and comments suggest a high risk of negative mental health effects, including depression and suicide. Most of these videos are not produced by experts such as dermatologists. The authors suggest this misinformation could lead to unjustified concerns and hinder patients from seeking necessary medication. They propose increased involvement of health professionals or artificial intelligence (AI) to combat misinformation on social media platforms [7].

The existing literature highlights TikTok's impact regarding the dissemination of reliable isotretinoin-related information, both personal and educational. Yet, significant gaps persist, notably the lack of research on TikTok users' perceptions and experiences regarding isotretinoin. This study aims to address this shortfall, providing comprehensive insights into attitudes, perspectives, and information dissemination.

## Materials and methods

On February 12, 2023, the video search for this study was initiated on TikTok, using the following hashtags in a single search query: #roaccutane, #accutane, and #isotretinoin. The retrieved videos were sorted by relevance, commencing with the most relevant video and proceeding sequentially. The inclusion criteria were as follows, and all criteria had to be met for a video to be included in the study: 1. The video was in the English language; 2. The video contained educational content, attitudes, or perspectives related to the use of isotretinoin in its audio content; 3. The video primarily focused on isotretinoin-related topics.

Exclusion criteria were as follows: 1. Duplicate videos or similar content from the same creator were excluded; 2. Videos that were predominantly visual with minimal or no audio content were excluded.

The selection process continued until 50 videos that fully met the inclusion criteria and avoided the exclusion criteria were identified for thematic analysis and agreed upon by both independent reviewers (AA and LH). To reach 50 videos, 102 videos were viewed in total (40 were excluded based on the exclusion criteria, and 12 were excluded based on not meeting the inclusion criteria). These videos were given anonymized TikTok numbers from one to 50 (TN1-TN50). The sample size of 50 was selected based on the principle of data saturation, as further videos were considered unlikely to provide additional insights into isotretinoin.

Data collection encompassed numerical information such as view count, likes, comments, shares, hashtags associated with the videos, video captions, and the identity of the video creator (e.g., doctor, patient, nurse, aesthetician, or pharmacist). Data extraction took place from February 19 to March 30, 2023, and following this, video transcription was done. Each reviewer then conducted an independent thematic analysis.

Following this, reviewers reconvened to discuss the emerging themes. A moderate level of inter-rater reliability was established. Subsequently, a joint coding manual was developed. A final combined thematic analysis was conducted, during which the identified themes were named and defined, and corresponding subthemes were appropriately integrated.

## Results

Quantitative findings

The selected videos were posted on TikTok between 13^th^ August 2020 and 13^th^ February 2023. Fourteen out of 50 videos had data available for the number of views, which approximated 607,700 views in total. Fifty out of 50 videos had likes, comments, and shared data, of which they approximately totaled 950,805 likes, 12,172 comments, and 13,195 shares. The data were accurate as of 30^th^ March 2023 (Appendix A). Data was collected on the demographics of those creating the videos in relation to their occupation or patient status (Table 1).

**Table 1:** Demographic data: roles of TikTok video creators

Role	Number
Patient	28
Doctor/Dermatologist	18
Dermatology nurse	1
Pharmacist	2
Aesthetician	1

Data were also collected on the frequency of each hashtag that appeared in video captions (Table 2). The results are summarized below.

**Table 2:** Frequency of hashtag appearance in TikTok video captions

Hashtag	Frequency
accutane	35
isotretinoin	32
acne	29
roaccutane	29
acnetreatment	11
fyp	7
skincare	7
foryoupage	4
skincaremyth	4
dermdoctor	4
trending	4
acnediaries	4
stitch	4
hormonalacne	4
dermnurse	3
acneskincare	3
dermbypark	3
acnescars	3
dermdoctor	3
skincareroutine	3
cysticacne	2
help	2
nosejob	2
duet	2
isotretinoinjourney	2
dermnurse	2
skintok	2
drdray	2
acnediary	2
spironolactoneskin	1
retinol101	1
roaccutanejourney	1
greenscreen	1
skinsafe	1
aesthetic	1
skincaremiracle	1
collagen	1
alixearle	1
aussie	1
sunburn	1
trending	1
spironolactone	1
acneskin	1
skincare	1
drsugaiskincare	1
mythbust	1
skinbydrazi	1
roaccutanejourney	1
dermreacts	1
roucutan	1
acnediaries	1
drnomzzy	1
skincare	1
sensitveskin	1
sideeffects	1
pores	1
skinhealth	1
isotretinoincheck	1
skincaremyth	1
acneskincareroutine	1
skincaretips	1
drdray	1
drdrayzday	1
drdrayrecommended	1
drsugaiexplains	1
sideeffects	1
dermatologist	1
acnejourneytoclearskin	1
darkskin	1
acnetreatment	1
londonskinclinic	1
roac	1
progress	1
darkskin	1
accnediary	1
londonskinclinic	1
acneproneskin	1
cysticacne	1
skinjourney	1
denverskindoc	1
acnejourneytoclearskin	1
pores	1
skinbydrazi	1
prescription	1
pharmacist	1
pharmacy	1
dermang	1
denverskindoc	1
skincaremyth	1
drdrayzday	1
accnediary	1
sensitveskin	1
i	1

Qualitative findings

The thematic analysis overall showed the emergence of eight themes and 28 subthemes. The results are summarized in Table 3.

**Table 3:** Thematic analysis of isotretinoin treatment: eight themes and 28 subthemes

1. Effectiveness and Benefits of Isotretinoin	2. Physical Side Effects and Challenges	3. Knowledge and Education About Isotretinoin	4. Psychological Side Effects and Impact	5. Personal Experiences	6. Treatment Process and Decision-Making	7. Awareness, Misconceptions, and Communication	8. Post-treatment Considerations and Future Outlook
1. Positive experience with isotretinoin; 2. Efficacy and benefits of isotretinoin; 3. Positive perception of isotretinoin	4. Physical side effects of isotretinoin; 5. Managing side effects; 6. Challenges of isotretinoin use	7. Individualized treatment and dosage; 8. Isotretinoin use, safety, and advice; 9. Educational value for patients, healthcare professionals, and students	10. Psychological impact; 11. Emotional side effects of isotretinoin; 12. Support system and encouragement	13. Personal experiences with isotretinoin; 14. Decision to start isotretinoin	15. Process of obtaining isotretinoin; 16. Information provided about isotretinoin's risks; 17. Medical monitoring and tests related to isotretinoin; 18. Evaluation for isotretinoin candidacy; 19. Ineffective prior treatments; 20. Need for dermatologist involvement	21. Clarifying misconceptions; 22. Lack of information and awareness on isotretinoin; 23. Importance of being informed; 24. Need for disclaimers	25. Reduced severity of acne post isotretinoin; 26. Limitations of Accutane in regulating hormones; 27. Post-Accutane health struggles; 28. Post-Accutane treatment considerations

Effectiveness and Benefits of Isotretinoin

The first theme that emerged was the 'Effectiveness and Benefits of Isotretinoin.' Within this theme, discussions highlighted the efficacy of isotretinoin in various aspects. Participants noted significant reductions in the appearance of scars, along with a shift towards positive experiences compared to prior treatments. This collective sentiment shows the profound impact of isotretinoin and its favorable perception among users. A testimony from TN29 encapsulates this theme succinctly: "I had so much scarring... Literally the one thing that has made my skin like this is Roaccutane. It's also called isotretinoin, and it is a godsend... the best thing I've ever tried."

Physical Side Effects and Challenges

A different theme, ‘Physical Side Effects and Challenges,’ elucidated the different struggles faced by individuals undergoing isotretinoin treatment. Discussions within this theme center on the management of isotretinoin-induced side effects, offering insights into the obstacles encountered during the course of this treatment regimen. This shows the need for management and integration of complementary therapies to mitigate distressing symptoms. Notably, participants commonly reported experiencing sun sensitivity, dryness, skin breakouts, and sore lips, underscoring the pervasive nature of these challenges. A reflection from TN47 encapsulates this theme, affirming, “I actually had to discontinue the medication a few months early due to the overwhelming intensity of the side effects.”

Knowledge and Education About Isotretinoin

Another theme identified was 'Knowledge and Education About Isotretinoin,' revealing crucial insights into the educational value of the videos. The content predominantly discussed the safety of isotretinoin usage. Numerous videos emphasized the pivotal role of information and guidance in ensuring the safe and effective utilization of isotretinoin. Key topics encompassed the significance of preventing pregnancy, understanding the mechanism of action, indications for use, dosage instructions advised by dermatologists, adherence to the medication regimen, and the correct method of consumption. A quote from TN20 encapsulates this sentiment: “Don't drink alcohol; it goes through the liver just like Accutane and can raise your liver enzymes and potentially lead to damage... It's important that Accutane is only taken by the person who it's prescribed for.”

Psychological Side Effects and Impact

Another theme emerged as 'Psychological Side Effects and Impact'. Discussions centered around the profound emotional and psychological toll experienced by individuals undergoing treatment. While some reported increased self-confidence as their acne improved, others endured heightened emotional distress and psychological symptoms. One participant recounted experiencing a 'depressive psychotic' episode, highlighting the severity of the impact and significance of supportive networks comprising family, friends, and healthcare professionals. A poignant reflection from TN28 encapsulates this theme: “As a month went on, I noticed that my mental health started to deteriorate really bad, and I actually started to go into quite bad depressive episodes. I found myself binge eating quite a lot to suppress my moods and try to make myself feel better, and I was sleeping a stupid amount, I'm talking like 13 hours a day... I also started to struggle with psychotic symptoms when I was driving and things like that; I would see things that weren't there, I'd smell things that weren't there, things like that.”

Personal Experiences

A different theme, ‘Personal Experiences,’ sheds light on individuals' unique journeys. Participants shared insights into their decision-making processes regarding treatment initiation and recounted their encounters with various therapies. They emphasized the numerous steps involved in accessing treatment and articulated nuanced perceptions of their skin's transformation pre-and post-treatment. However, personal narratives exhibited diversity, with some individuals reporting minimal observable changes in their skin throughout the treatment duration. A poignant reflection encapsulating this theme comes from TN22: “I'm coming from a place where this all happened to me personally, and on top of that, Accutane did nothing for my skin... sadly I am not the only one that has that story.”

Treatment Process and Decision-Making

Another theme identified was ‘Treatment Process and Decision-Making.’ Contributors extensively discussed their experiences with prior ineffective treatments, often resorting to multiple rounds of antibiotic regimens before considering isotretinoin as a final option. Moreover, a significant aspect highlighted was the rigorous evaluation process preceding treatment initiation. For instance, individuals of childbearing age underwent mandatory pregnancy tests due to the potential adverse effects of isotretinoin on fetal development, showing consideration and awareness of potential risks integral to the treatment decision-making process. A poignant quote from TN34 encapsulates this sentiment: “They’ll talk to a dermatologist, the dermatologist will send you a leaflet in the post, and you'll obviously get to read over all the symptoms and get to read over all of the health risks involved with Accutane because there are a lot. There are a lot of health risks.”

Awareness, Misconceptions, and Communication

The penultimate theme, ‘Awareness, Misconceptions, and Communication,’ explored prevalent misconceptions and the significance of effective communication. Participants highlighted common misconceptions, revealing doubts about treatment efficacy stemming from insufficient information and awareness, which can significantly influence individuals' ability to make informed decisions. An excerpt from TN45 encapsulates this sentiment eloquently: “Now, it does have its side effects, and most are quite manageable, but because of that, it gets a bad rep online, and often patients dismiss it based on what they've heard from friends or online.”

Post-treatment Considerations and Future Outlook

The final theme identified was ‘Post-treatment Considerations and Future Outlook’. Key discussions revolved around the multifaceted approach required to manage acne recurrence, particularly in cases involving hormonal imbalances. Participants emphasized the importance of optimizing treatment outcomes by adopting personalized skincare routines and considering additional medications tailored to individual medical histories, all aimed at maximizing treatment efficacy. A quote from TN39 encapsulates this sentiment: “This means that my patients with hormonal acne still need something to treat their acne even after a round of Accutane. What could that something be depends on how badly their hormonal acne recurs. Options for hormonal acne depending on your risk factors include oral spironolactone, topical spironolactone ... or possibly birth control pills.”

For a more detailed insight, refer to Appendix B, which presents a more extensive thematic analysis. This appendix includes a substantial, though not exhaustive, selection of quotes from the transcripts to illustrate each identified theme and sub-theme comprehensively (Appendix B).

## Discussion

To the best of our knowledge, this study is the first to conduct a thematic content analysis of TikTok videos on isotretinoin use, examining perspectives, attitudes, and educational value. These findings emphasize social media platforms' role in disseminating health information about dermatological treatments like isotretinoin. However, they highlight the need for dermatologists to monitor social media trends to understand patient perspectives [4] and address any misinformation that may discourage treatment [5-9].

Patients are increasingly turning to social media, including TikTok, a platform with over two billion downloads, for health-related information [8,9]. TikTok's predominantly young user base, with 63.5% under 24 years old, highlights the significance of addressing health concerns like acne in this demographic [6,8,10]. Such platforms facilitate education and Q&A sessions, enabling users to access information efficiently. Notably, social media serves as a rapid and effective medium for medical professionals and laypersons to share healthcare insights. With over 80% seeking medical information online and 90% of Gen Zs utilizing social media for medical advice, its role in healthcare dissemination is pivotal [11,12,13].

The diverse narratives of 'Personal Experiences' shed light on individual journeys with isotretinoin, revealing varied perceptions of treatment outcomes and skin transformation based on different underlying motivators to share one's experience online. This is in alignment with prior studies exploring the motivations for user engagement and content creation on TikTok [14].

Within our study, the significance of positive testimonials reinforces the efficacy of isotretinoin, particularly in addressing treatment-resistant acne. The theme of 'Effectiveness and Benefits of Isotretinoin' emerged, displaying the positive impact of isotretinoin on scar reduction, skin improvement, systemic benefits, significantly enhanced self-esteem, and improved mental well-being [4,7]. Users expressed satisfaction with the efficacy of isotretinoin, highlighting its transformative effects on their skin [15], as evidenced by testimonials like the one from TN29. This observation resonates with previous research, which contributes to the discourse on the portrayal of isotretinoin's mental well-being benefits on TikTok [4,7,15].

In contrast, our study also reveals users' experiences regarding the 'purging phase,' characterized by worsened acne prior to improvement. Despite its transient nature, this discomfort involves physical and psychological manifestations. Particularly, detrimental effects on well-being and self-perception correlate with heightened levels of anxiety among TikTok users [1,7]. The analysis of 'Psychological Side Effects and Impact' explores the emotional repercussions of isotretinoin treatment, ranging from self-confidence to severe psychological symptoms such as depressive episodes, psychotic manifestations, and suicidal ideation [4,7].

Our study’s theme of ‘Post-treatment Considerations and Future Outlook’ highlights the significance of tailored skincare regimens and supplementary medications in addressing acne recurrence following isotretinoin therapy [4,16]. This parallels prior research examining health promotion discourse on TikTok, highlighting its potential for health communication [17].

Furthermore, the theme of 'Knowledge and Education about Isotretinoin' underscores the pivotal role of high-quality educational content disseminated on TikTok, particularly in promoting safe usage, including pregnancy prevention, dosage guidelines, and adherence to treatment protocols [4]. Studies delving into the influence of TikTok on dermatological discourse shed light on its potential as a vehicle for education and patient engagement [8, 18]. These observations corroborate existing literature, accentuating the criticality of ensuring the quality of health information disseminated via social media channels such as TikTok [19]. While recognizing this promise, researchers caution against the prevalence of misleading and potentially harmful content circulating on the platform [11].

The discourse surrounding 'Awareness, Misconceptions, and Communication', focuses on the impact that misinformation and limited awareness can have on treatment decisions, emphasizing the necessity of effective communication aimed at dispelling misconceptions, particularly regarding isotretinoin [4, 6, 20]. Insights gleaned from studies examining negative user interactions with short-form video content on platforms like TikTok [21] suggest that the time-constrained nature of such videos is a factor. However, the dynamic nature of such platforms provides dermatologists with invaluable insights into patient perspectives and knowledge [12, 22]. Being vigilant on emerging trends and leveraging trending content could enable dermatologists to not only combat misinformation but also promote accurate and beneficial information, thereby fostering informed decision-making among patients [23].

To combat misinformation about isotretinoin on social media, healthcare professionals can create targeted educational content to address common misconceptions and offer evidence-based guidance. Artificial intelligence, in the form of bots, for example, could be used to identify and flag misinformation, particularly regarding mental health risks, and direct users to reliable sources provided by healthcare professionals. Artificial intelligence can employ natural language processing to identify misinformation using relevant keywords and post-corrective responses [24] and could be developed to monitor social media for emerging misconceptions. This allows timely intervention to prevent the spread of inaccuracies before they become pervasive. Collaborations between dermatologists and influencers can further amplify reliable health messages, ensuring they reach wider audiences effectively.

This study employs a rigorous methodology, utilizing thematic analysis of TikTok content to showcase our findings. However, limitations include potential investigator bias in video selection, which could influence findings, and hashtag search strategy methodology, which may not capture all relevant content. Additionally, the exclusive analysis of TikTok videos limits the breadth of the study by excluding other social media platforms that might offer a wider range of opinions and perspectives. Furthermore, it is important to acknowledge that TikTok is banned in some highly populated countries such as India, North Korea, Nepal, Afghanistan, Syria, and Somalia, with China using a domestic version called Douyin. This limited accessibility may not fully represent the diversity of experiences and views, thus limiting the extent of this study’s generalizability to global populations.

## Conclusions

TikTok enables users to share varied experiences and views on isotretinoin treatment, revealing the intricate interplay between personal narratives and social media's influence. This study identified eight main themes and their sub-themes, offering insights into isotretinoin usage and the need for reliable health information online. Future research should investigate strategies to promote accurate health information on TikTok. Evaluating long-term effects of health education from TikTok regarding the impact on treatment adherence and patient outcomes is essential. In conclusion, accurate health information and online education are crucial. They foster important discussions, supporting better patient care and improving acne management.

## Appendices

Appendix A 

**Table 4:** Number of views, likes, bookmarks, and shares of TikTok videos

TikTok Number	Views	Likes	Comments	Shares
1	27,000	1083	171	10
2	N/A	1359	18	2
3	1700	16	8	1
4	N/A	5137	147	41
5	N/A	13,300	271	574
6	N/A	67	27	1
7	82,500	1066	36	19
8	N/A	450	19	3
9	13,900	883	17	5
10	N/A	111	22	12
11	N/A	91	34	5
12	N/A	6720	131	362
13	1825	18	6	1
14	N/A	29	11	2
15	882	116	0	1
16	N/A	446	13	10
17	1638	41	17	1
18	N/A	194	2	2
19	N/A	3699	83	12
20	N/A	26,800	622	730
21	N/A	13,500	335	168
22	N/A	522	43	47
23	171,100	9320	127	257
24	N/A	214,200	1928	6628
25	N/A	4485	103	48
26	N/A	514,300	6072	4799
27	43,700	3229	53	30
28	N/A	11,300	99	43
29	N/A	41,100	225	877
30	N/A	39	12	2
31	N/A	38,400	222	752
32	N/A	2262	48	56
33	N/A	48,400	518	2243
34	N/A	2857	53	27
35	10,700	134	12	12
36	N/A	8352	246	219
37	21,300	852	39	9
38	N/A	402	0	9
39	N/A	1398	22	18
40	N/A	103	5	3
41	748	42	13	2
42	N/A	140	0	1
43	1998	31	0	1
44	N/A	136	27	5
45	105,100	4332	139	49
46	N/A	111	5	3
47	N/A	599	21	3
48	N/A	9171	198	154
49	39,300	975	14	15
50	N/A	88	8	2
TOTAL	670,700	950,805	12,172	13,195

Appendix B 

**Table 5:** Thematic analysis data representing transcripts grouped into respective themes and subthemes

Theme	Subthemes	Quotes
Effectiveness and Benefits of Isotretinoin	Positive experience with Isotretinoin	“I know it's a drug that gets quite alot of backlash, but I had a really positive experience with it and that's why I kinda wanted to share sort of my journey on isotretinoin aka Roaccutane, aka a massive dose of vitamin A…single-handedly one of the best things I've ever taken." - TN1 “I had so much scarring...Literally the one thing that has made my skin like this is Roaccutane. It's also called isotretinoin, and it is a godsend... best thing I've ever tried." – TN29 "Hello, so it may not look like it - but today I've woken up without any spots for the first time in over a year. I still have a lot of scarring, but that will go with time, I've been on Roaccutane for 4 months and for part of that I was also taking erythromycin." -TN37 "I suffered from severe acne and I was also placed on Roaccutane...You will make it through to the end, I promise you will. I did, and I'm sure you will." – TN41 "I'm thankful for Roaccutane and it's the only thing that's worked for me." – TN44 "I went on a medication called Roaccutane... after being on it - I think it was like, 6 months, this is my skin, and I couldn't not be happier." – TN47 "Today is officially 1 month since starting Accutane which is quite exciting. My skin today, it's looking quite good at the moment which I am quite impressed with." – TN50
	Efficacy & Benefits of Isotretinoin	"It was full blown metamorphosis, and bar the odd bit of hormonal acne, like here and here, it stayed that way. My favourite part is the skin texture. it gave me 10/10." - TN1 "So, when you're taking isotretinoin, or otherwise known as Accutane or Roaccutane in the UK, you are getting a systemic benefit as a result of taking the oral medication." - TN3 "It is now week 15, I think um, and my skin is kind of clearer um, but like you can still see stuff so annoying. These are freckles. no need to get off that. My skin is just not gonna look smooth smooth. But there is still spots...I'm getting around here. I'm getting like the odd one on my back and like bum and legs still, that's annoying." - TN6 "My skin just always seems so much glowier now." - TN7 "As you can tell like, on my cheeks, it's basically scarring, but it's gone down a lot, like the flaring is going down a lot." - TN9 "I took it for about 10 months, my skin was perfect, it was all good... my skin was amazing, everyone was commenting on my skin, it was glowy, it was baby face, it was beautiful, it was amazing, it was vibrant, it was everything I ever want...and then my acne was gone completely, my skin was glowing, I've never had such glowy skin until since before I was I don't know how old, um my skin was amazing, everyone was commenting on my skin, it was glowy, it was baby face, it was beautiful, it was amazing, it was vibrant, it was everything I ever want." - TN17 "This video is absolutely amazing, watch her skin transition from the beginning of this video to the end of this video…In just a few months you see her skin is already doing much better...it's a cure for acne for about 80% of people, and look at her skin, it looks amazing…Nothing you find at CVS, Sephora, Ulta, Walgreen's is even going to touch this form of acne." – TN26 "Guys it's month 3 of Roaccutane, look at my skin in September oh my god it was so sensitive and broken, so raw, and now month 3 on 60 mg, look at the difference, how exciting ahhh! Still getting under the skin spots." – TN35 "I do feel like it was the right decision for me." – TN42 “This was kind of like the first photo where I was like oh my god. Like I feel good, and I almost like forgot what I had like, looked like. Because I was so, when I took a photo of my face and when I would look at myself in the mirror, all I would see was acne, not my face…. it's just like such a big difference in the ways it's like. The textures are not as bad, and like I just don't have the breakouts anymore... it is helping me mentally so much right now” – TN44 "If you have a lot of oil glands on your nose that is making the nose thicker, then accutane will shrink down those oil glands and slim down your nose." – TN48
	Positive Perception of Isotretinoin	"I always say this can be a life changing medication, don't worry about the fear mongering on the internet, trust your doctor." - TN19 "If you have bad acne, Accutane is an amazing drug." – TN23 “You're about to watch an Accutane glow up, now this is by far the most effective treatment that we have for acne…The results can be lifechanging." – TN24 "I was very nervous to try Accutane because I only heard horror stories about it, but the more I started asking around, people I immediately knew in my sorority and like, at college and stuff, it seems like it helps them. So, I thought I'd give it a shot." – TN27 "finally, finally, finally, they prescribed me Roaccutane...I'm excited; I'm anxious about the journey...I've been waiting for a few years for this moment...So, I'm just happy to finally be here." – TN30 "Accutane is an amazing treatment for acne” -TN33 "The side effects are bad but I'd also say it's worth it." – TN37 "I'm really pleased to see that she had a great result from the Accutane." – TN40 "So, this is my couple months progress, so obviously I am thankful for Roaccutane." – TN44 Stitch: "a free nose job back in 2016 - I was like yeah, we were on accutane, that shit shrinks your nose”…Dr Azi: "In fact, dermatologists will start patients after a nose job on accutane to help slim down the nose." -TN48
Physical Side Effects and Challenges	Physical side effects of Isotretinoin	"My skin was peeling off, my lips were so dry, my nostrils were so dry, I kept going pink every time I'd laugh... and I had really bad purging for like 2 months." - TN1 "Signs that you're on Roaccutane. Your skin is shiny, I feel like a disco ball. You continue to get break outs, they're cystic and painful. Your lips start to get really dry and as you open your mouth you get cracks in the side and you resemble the joker, fun.” - TN5 “But I did have to have a little few weeks of not taking the tablets because I kept getting migraines like a lot like for a solid month. I kept getting like a constant pain kinda like dedicated to this eye like round here and sometimes it would get a lot worse and not, so I like stopped taking the tablets. Then my eye was still hurting for the week, but the end of the week it kinda wasn't. So, I was like, okay, coincidence or is it tablets. Then I start taking the tablets again and it came back really bad and I was like, hmm, maybe the tablets. So, I went to the opticians and there isn't anything actually wrong with my eye. So, since then I've been taking the tablets but at the lower dose.” - TN6 "My skin's feeling alright, I've had a couple wee cysts pop up recently, but the pain's not been too bad” - TN7 "I feel like I can sort of start to feel my lips starting to dry up a little bit but they have not been bad. I’ve been waking up during the night and my mouth has been so dry, having to drink loads of water during the night. I wake up in the morning and my mouth is so dry and my throat, so that’s everything definitely starting to dry out. I can feel it like actually starting to purge everywhere, like I can feel parts where it's all coming out like here, and here [points at forehead]. Usually it is mostly just on my cheeks, so for it to be all breaking out here [points at forehead] – it must be definitely just starting to purge." - TN8 "I've also only had one nosebleed, which compared to my last time, is really good for me." -TN9 "Actually, it has significant side effects...Your skin can get a bit dry and irritated when you are on it." - TN14 "Accutane can make you sunburn quickly and unexpectedly." - TN18 "Now when I started on 30 milligrams, I started noticing problems with my heart, but my dermatologist and a few doctors told me it was nothing related to Accutane, and then some doctors told me that it was Accutane...A quick disclaimer as well, everyone's body reacts differently to different meds, and everyone is different, so my experience with Accutane is not going to be the same as your experience or the next person." – TN28 "Accutane, we know, it completely dries out your pores, it dries out your skin...Because it works in that way, patients on Accutane also experience dryness everywhere...They get flaring of their eczema, they can also get dry lips, drying inside of their noses and also very very dry eyes." – TN31 “Yeah I take them with my vitamins at night, but the worst are the veins popping out of my skin, like everywhere all-round my body, I look like hulk hogan, I'm hoping this will go at the end of treatment" – TN35 "Okay fair warning that this photo is really gross because this is when my cysts are sort of coming out of Roaccutane, and um yeah, I would literally move my face and that would burst. Ew this is sort of more from just the purging, where it would be like, lots of whiteheads, which I could deal with because I knew it was like coming out." – TN44 "I've just noticed that my lips are even worse than ever, they're not even dry it's just that the skin won't stay on my lips, like they are just completely peeling off the whole time…Another thing I am noticing is dry throat... I don't even know if that is a side effect, but it is happening. I am noticing as well that my hair is not getting greasy at all which is great. Other than that, the skin on my arm and my hands is a bit flaky." – TN46 "My hair started falling out, I went into like a depressive psychotic episode, I really don't know. My lips went so dry, my nose went so dry." – TN47 "The second one is nauseousness, I had this and as females we already get semi-nauseous when we're on our periods...you're gonna get nauseous because of your period, and you're gonna get nauseous because of the treatment, so you're just nauseous all-round.“ – TN49 "Your skin is really sensitive...you're just tired all the time and ache." - TN50
	Managing Side Effects	"I probably just need to drink lots more water, because I'm struggling with that right now, especially as an athlete." -TN9 "Your lips and skin might get excessively dry and sensitive to sunlight, lip balm and moisturizer,might help." - TN10 "She's gonna need aloe vera, Ibuprofen can help with the pain...Lots of water is important while on Accutane." - TN18 “So make sure that when you are outside you have plenty of sunscreen." - TN20 "Your lips are going to be chapped and dry. That's to be expected! So, carry around Aquaphor, Vaseline and if that doesn't cut it try Dr dan's Corti balm or something like Lipster…Stop all of your other acne treatments and just use gentle cleansers and moisturizers. For dry eyes, I like Systane. If you have a nosebleed or dry nose, put a little bit of Vaseline or Aquaphor in your nose before you go to bed. Those are always the big ones I recommend to my patients." – TN25 "I've read up on the side effects, I know it's not gonna be easy, um but I'm prepared for it.” – TN30 “I typically recommend sleeping with a humidifier, especially during wintertime, and then using a Q-tip to apply a thin layer of Vaseline to coat the inside of your nose every day. For dry eyes, I do recommend trying to cut down on contact lens use that could potentially irritate my patient's eyes and lead to more dryness. I also recommend using refresh tears to re-wet their eyes constantly throughout the day.” – TN31 "I find that my skin hasn't been too dry, I use the Cerave hydrating moisturiser and face wash, and I find that their pretty decent." – TN37 "Keep your skincare simple and not to aggravate, keep your skin hydrated and moisturized." – TN41 "I've noticed that lip balm is not moisturising enough to deal with the cracked lips you get with this treatment...So, make sure if you don't like the LANEIGE one, find another lip mask that's just as moisturising or else it's going to be hurting, and you're going to have bleeding on your lips." – TN49 "Also, a hat, essential if you are going out in the sun because your skin is really sensitive… drink loads of water because you’re just tired all the time and ache." – TN50
	Challenges of Isotretinoin use	"So, I'm getting a little bit paranoid...Usually, I would come on my period like the 2 every month, obviously it's like 9th...it's weird but sometimes that happens." - TN2 "I need to stop taking them in like early Feb because I'm going to hot countries and you're not meant to be in the sun." - TN6 "My lips are kicking me in the butt because I just keep picking at them, because you can literally peel off the skin and it is so satisfying." - TN9 "There is a purging phase where your acne will get a lot worse... it got worse before it gets better." - TN27 "I've had to sign this form that I was not planning to get pregnant in the next 6 months...I was not going to have sex for the next 6 months because I refuse to go on contraception just because of the side effects...Um, yeah, there's loads of paperwork…So, the day is Sunday the 7th of August, so this is like peak summer...obviously with Roaccutane you have to be really careful with the sun exposure." – TN30 “So, it wasn't too bad, and like I was managing that. I would get over it. Some days it would affect me, some days it wouldn't…and that definitely gave me a purge. Sorry. Didn't love it but I know it would get better, so I stuck with it." – TN44 "Especially where my face is recovering and stuff because of covid, the skin is just not staying on my lips even though I am moisturising like constantly." – TN46 "I actually had to finish the medication a few months early because the side effects were too intense." – TN47
Knowledge and Education about Isotretinoin	Individualised Treatment and Dosage	"I've got my dermatology appointment next month, and this is supposed to be my last month but I have a feeling I'll probably get kept on it a little bit longer, just cos you know there's quite a bit left, but we'll just wait and see." - TN7 "I think it depends on your height and your weight. So, when I first went um they took my weight, took my height and then um came up with like a total sum of how much um Roaccutane I'll be on. So, I think mine was like 7500, could be wrong, something similar, like around that, and then they work out how much you would need. But they started me out on 20 mg, and then increased it to 40, increased it to 60, the 60 I didn't tolerate very well, so they dropped me back down to 40 and then I just stayed on 40 for the duration. So yeah, I'm pretty sure it does change for um each individual, on like the height and weight but again I'm not a professional, so don't quote me. And yeah, I think I remember seeing a TikTok of someone saying that they were on 80mg a day which I would never have, like been able to cope with that, um like I said I couldn't even tolerate the 60, so my dermatologist just dropped me back down to the 40, and I stayed on that for the whole time." - TN15 “A lot of people got like 100 milligrams things like that, but they want to slowly build me up, so I started on 20 and I went up to 30 on month three” – TN28 "It's a medication that's taken orally, for about 6 to 9 months on average, sometimes more, sometimes less, we dose it based on weight, " – TN45
	Isotretinoin use, safety & advice	"Using retinol or tretinoin ... they are all derivatives of vitamin A ...You do not need to use them both when you are taking oral isotretinoin." - TN3 "It's also extremely important not to get pregnant whilst on this medication and one month after stopping this medication and this is because isotretinoin can harm an unborn baby. And listen carefully, this is vital information whilst taking this medication if you have depressive thoughts or have thoughts of self-harm. then stop this medication and contact your doctor straight away.” - TN10 "We shouldn't be injecting through skin that's inflamed or has areas where there are pockets of infection...All this is part of the consultation...we have to understand the medical conditions and how these conditions affect the body." - TN14 "Don't drink alcohol, it goes through the liver just like Accutane and can raise your liver enzymes and potentially lead to damage...It's important that Accutane is only taken by the person who it's prescribed for." - TN20 “You definitely cannot get pregnant while taking it…You do need to take medications by mouth to help heal this” – TN26 "As a dermatologist, here's a sample skincare routine that I recommend for my patients on Accutane for the morning, first step is to cleanse your face with a mild cleanser and avoid salicylic acid or benzoyl peroxide. Step 2 is to hydrate your skin a lot with moisturizers with ceramide/niacinamide or hyaluronic acid. And step 3, super important, sunscreen that leaves your skin hydrated. Nighttime super simple: step 1 hydrating cleanser, and again step 2, lots and lots of moisturizer, and finally, plenty of Vaseline for the lips because Accutane dries out your lips a lot." – TN32 “Here's some skincare tips: don't use any acne products while you're on Accutane, use a gentle cleanser and an SPF day cream and the same gentle cleanser and moisturizer at night. Your eyes and lips will get very dry, use Aquaphor on your lips and refresh tears for your eyes, your nose can get very dry and even cause nose bleeds, so use a nasal saline spray“ – TN33 "Want to know a reason why Accutane failed? You took it on an empty stomach, Accutane otherwise known as isotretinoin has to be taken with food, ideally food that has a little bit of fat in it, something like avocado or peanut butter or almond butter if you're all about that life." – TN36 "3 top tips for starting Roaccutane. 1: use a lip balm with SPF... 2: moisturise day and night... 3: try and drink as much water as you can." – TN38
	Educational Value for Patients, Healthcare Professionals & Students	"You are getting a systemic benefit as a result of taking the oral medication." - TN3 "Isotretinoin can start to work from a week to 10 days." - TN10 "Isotretinoin is an oral medication we use for moderate to severe acne. While on this medication your sebaceous glands do shrink and this is proven with pathology findings. If your nose is thicker with a lot of sebaceous glands, then yes, those glands will shrink, but not to a big noticeable degree. This JAMA paper shows that sebaceous gland sizes do decrease after four months of treatment." – TN12 "I thought I would record my journey and give a good insight and good reflection for myself to see the difference really." – TN13 "It's what we call a vitamin A derivative, so it's derived from vitamin A... the pilosebaceous subunits in the skin which produce the sebum and the oil, etcetera, which as they are, that's the problematic subunit in the skin that results in acne. So, what this product does is it warps the pilosebaceous unit when you take it as an oral tablet and then it makes it dysfunction so it can't function as well, thereby removing the cause of the acne in terms of your skin...So, if you're going to someone, they should understand why you have acne, understand what isotretinoin actually does, and understand any risks involved in that treatment and then discuss that with you." - TN14 "Don’t get tattoos or piercings. When you are on Accutane, you don't heal quite the same way. So, you are at risk for infections or granulomatous reactions from a piercing or elective procedure." - TN20 "So, let's watch, and then just to give you a background with Accutane, it's the brand name for isotretinoin. And Accutane is an oral retinoid, and the main thing you'll hear about it is that it is contraindicated if you are pregnant because it can cause birth defects." - TN21 "Accutane or isotretinoin is an oral medicine that we use to treat moderate to severe acne and it works by shrinking your oil glands and decreasing oil gland activity " – TN23 "Is there any educational value to these videos for patients, healthcare professionals, and healthcare students?" – TN26 "I thought I'd do this video to literally explain why I had to come off Accutane, like my experience…If you guys have any further questions, feel free to ask." – TN28 "So, this is I guess probably day one of my skin diary." – TN30 "But for the old school classic isotretinoin otherwise known as Accutane, has to be taken with food in order for it to get into your body and work." – TN36 “I always ask if their acne happens to be hormonal acne. Now how do you know, hormonal acne is acne that tends to appear around their chin and jawline region.” – TN39 “I assure you, even to my masters when I did my master's degree, I did the whole therapeutic profile of Roaccutane or isotretinoin, and my take-home message to you is that it will get better, I promise you it will." – TN41 “I reserve it for patients who have failed other acne treatments, or if I see early signs of scarring or current scarring”- TN45 "I thought I would do a couple of tips if anyone is starting Accutane or anything." – TN50
Psychological side effects and Impact	Psychological impact	“It was bothering me a lot...he was like, really for you, for someone who's got recurring issues with acne, and it's psychologically impacting them, they should just put you on one short cycle of isotretinoin” - TN1 "I don't think the mental health stuff is to do with the tablets because the mental health has been decent." - TN6 "I'm not sure how much that's me being paranoid that I'm getting skin damage already because you're supposed to stay out of the sun as much as you can…Now I'm worried that I'm like giving myself skin damage because of that." – TN13 "As a month went on, I noticed that my mental health started to deteriorate really bad, and I actually started to go into quite bad depressive episodes I found myself binge eating quite a lot to like suppress my moods and try to make myself feel better, and I was sleeping a stupid amount and I'm talking like 13 hours a day...I also started to struggle with psychotic symptoms when I was driving and things like that, I would see things that weren't there, I'd smell things that weren't there, things like that." – TN28 “My mood has also been absolutely fine, I think that was one of the main things I was worried about going onto Roaccutane as everyone says it makes you really depressed but even during the pandemic, I've been okay so if you're worried about that I wouldn't let that be the thing that puts you off.” – TN37 "I went into like a depressive psychotic episode... after being on it - I think it was like, 6 months, this is my skin, and I couldn't not be happier." – TN47
	Emotional side effects of Isotretinoin	"I have been extremely moody as well." - TN2 “You also experience mood swings where one minute you feel fine & you're great and next minute, this happens {switch to pre-recorded video of her sobbing} this is so dumb, there's nothing even wrong.” -TN5 "When I see videos like that, it absolutely breaks my heart because I was there." – TN27 “I found myself binge eating quite a lot to like suppress my moods and try to make myself feel better” – TN28 "My side effects: I'm very emotional” – TN35 "This medication does have a certain number of side effects that can affect you emotionally." – TN41
	Support System and Encouragement	“I just want to say thank god I had a good support system, like my family, my mum was saying stick with it, it'll work, just give it time, my friends were saying your skin looks so good now, just remembering hearing those words in my heart, just it like fluttered." - TN27 "If you need to DM me at any stage to give you information and advice, then I'm more than happy to do so." – TN41 "If you have any other questions or things that you would want me to update you on during this process let me know." – TN43
Personal Experiences	Personal Experiences with Isotretinoin	"So, this was the day I went to the GP to get it sorted, so that was my skin day 1. This is day 2, not much change. Day 3 I noticed I'm getting a bit itchy and a lot more breakouts, I think it's called like purging when your skin just breaks out more, then it just got a little bit dry, this was Saturday, so you can see how dry it got there, my lips were starting to get very dry at this stage. This is today, which is Monday, so a week today, terrible lighting I'm sorry, and so yeah, this is my lips, and they are bad, but I'll keep you updated " - TN4 "I still feel okay otherwise, so yeah doing good, I'm just excited for this to all be over and have nice skin." -TN8 "Right this is going to be my first entry just of me recording my journey on Roaccutane or isotretinoin however you say that. Basically really really strong stuff to try to get rid of acne…I've been taking it for about a week now, I forgot to kind of do like the actual start but there hasn't been too much difference in my skin really…My skin hasn't been too bad in the past couple of weeks anyway I think because of the sun, I don't know, where I am with my hormone cycle maybe…But again, once I stop, they come back here's a few photos of what my skin looked like before…and there are many, many side effects…So yes, here is the first bit of my skin right now and then I'm gonna post another video explaining all the side effects…right now I'm not feeling any too much except I've got a red tinge to my skin kind of…I have been wearing suncream a lot except if I'm gonna be in the sun for like 30 minutes, then I can't be bothered to sun cream my entire body I tend to just do my face …But yes, this is week one." – TN13 "Before I went on Roaccutane, I remember waking up and being really worried about what I was going to be dealing with on my face. My skin was inflamed and angry, and it hurt...I look back on that [snapchat memory] and I see how sad I was, and my skin genuinely hurt. After 8 months on Roaccutane, and it's now been 4 months off, here is the result." - TN16 "So then many years later, fast forward to a year ago, um I about 2 years ago, I stopped the pill... and a lot of other things, but I'm gonna get into that another video, so I stopped the pill, I started doing treatment for all my health issues and politics to go with reasons, a lot of other more serious health issues...And that's when my acne came back and after a lot of other treatments trying to tackle it, we realized ...it wasn't going to go away, and that's when I decided to go on Accutane and my dermatologist agreed and prescribed me with Accutane again...And then literally I'm gonna take you um 5 months ago, I basically stopped Accutane cause obviously it was done, it was fine, whatever, and then a month ago, or a month and a half ago, I started getting acne again because this girl is too hormonal and I have polycystic ovaries and it's just not gonna go away." - TN17 "I'm coming from a place where this all happened to me personally, and on top of that, Accutane did nothing for my skin... sadly I am not the only one that has that story." – TN22 "So as a lot of you know, I struggled really badly with cystic acne, and so to treat that I had to go on medication called Accutane or also known as isotretinoin…I've now been off Accutane for 7 months, and I have been very lucky, I've been very lucky that basically my acne hasn't kind of made a reappearance…everyone's body reacts differently to different meds, and everyone is different, so my experience with Accutane is not going to be the same as your experience or the next person…This was a really rare side effect just to let you know, so that's what my dermatologist told me, but it started to get quite bad, I rang my dermatologist and they basically explained that I have to come off the meds, and I can't complete the course. So, this is what happened, but they did let me know obviously when you finish Accutane early that you're more like, your acne could like come back and stuff like that” – TN28 "So, although my skin isn't perfect now, I wanted to make a video talking about my experience with Roaccutane as it's something I get asked quite a lot in my comments." – TN37 "Hi everyone, so this is just my six-week update on Accutane...I haven't really noticed much changes this kind of time... but other than that no changes so I will update you again when I know more” – TN46 "I had to finish the medication a few months early because the side effects were too intense." – TN47
	Decision to Start Isotretinoin	"I have debated going on this tablet for so so long, because I know it can be so severe but I have tried so many lotions and potions I've tried so many prescription tablets and creams and just nothing has worked so yeah I decided to go on this tablet" - TN4 “Turning point for me was when I rushed at Alabama, the week I rushed my skin broke out so bad, it was severe acne. It was like my pimples, had pimples all over my face. I had a massive one on my chin that I had to get a cortisone shot because it was inflamed, and I had it for 2 weeks after I rushed. Also, around that time was when I started going to the gym, that's kind of where my gym journey started. But I was always self-conscious because I would go in the gym with this massive, red, volcanic face, and I remember leaving my workouts, like looking in the mirror just being so upset because I felt so ugly. There's this really sweet girl who was talking to me, after one of my gym sessions, kind of talking about Accutane and everything, so I was just telling her, like I'm done with my skin, I hate it, and I ended up going to see a new dermatologist, and that new dermatologist prescribed me Accutane.” – TN27 “I have been struggling with it for 10 years. Uhm I've been on four or five types of antibiotics, topicals, I've tried herbal remedies, uhm I've tried diet change, so this is really the last resort at this point.” – TN43 "This is day 1 of when I decided to go on Accutane because I was like, I just couldn't mentally deal with the skin anymore." – TN44 " My skin used to be dreadful! This is my forehead a year ago, look at the difference, I mean I still have a little bit of scarring, I don't know if you can see, like it is a little bit textured, but it's like nothing compared to that. And like about a year ago, this was my chin, I feel like my chin was the worst, and now like I really don't ... I get a little break out but it's really nothing. So, you might be asking what did I do to clear my skin up, so this was about four months into my acne treatment. I went on a medication called Roaccutane." – TN47
Treatment Process and Decision-Making	Process of obtaining Isotretinoin	“So I went on the combined pill, also known as the Yasmin pill, I was on that for 4 weeks and then I got to start um Accutane" – TN28 "A step by step on how to get Accutane. First of all, go to your normal GP, go to your normal doctor. Don't wear any makeup and bring photos of previous breakouts. Tell them that you're having issues with your acne. Tell them if it's painful, if it's really red, if you're getting loads of cystic acne. Obviously, they will be able to see it and be able to say "um -alright obviously you do need some help". They'll ask if you've been on antibiotics, they’ll ask if you've been on the pill - am talking for women in this case. And if you have and none of that worked, they'll obviously be like Accutane is a last resort drug, you seem like you're up for it…There are a lot of health risks. And then again, if you really want to do have this, you can agree by signing a medical form and then you need to have a blood test, your blood test will be fasting, and it will be to check your liver function, your kidney function and your lipids.” – TN34
	Information provided about Isotretinoin's risks	"Now, before I go any further, as effective as this medication is, it has some serious side effects." - TN10 “But it also comes with a lot of side effects and risks, so we don't take this medicine lightly." – TN23 "You cannot get pregnant on accident, well you can, but it's really dangerous and the medication is really dangerous and could be damaging to the foetus." – TN28 "They'll talk to a dermatologist, the dermatologist will send you a leaflet in the post, you'll obviously get to read over all the symptoms, get to read over all of the health risks involved with Accutane, because there are a lot. There are a lot of health risks.” – TN34 "Once we've kind of agreed that that would be the next step, all the things, they go through potential expected side effects." – TN43
	Medical monitoring & tests related to Isotretinoin	"But I did have to have a little few weeks of not taking the tablets because I kept getting migraines like a lot like for a solid month. I kept getting like a constant pain kinda like dedicated to this eye like round here and sometimes it would get a lot worse and not, so I like stopped taking the tablets... So, I went to the opticians and there isn't anything actually wrong with my eye... I went to, well my monthly appointment at the hospital. I told them and then they were like we'll refer you to an emergency appointment with the ophthalmologist there which already just received the appointment thing, and the emergency appointment is in January and they've also referred me to the Neuro team because one of the side effects, it’s like this, like a pressure thing in your head, like some form of benign thingy, Which I can feel - pressure in my head” - TN6 "You'll take a blood test before you start this medication and whilst you're taking this medication to check for side effects." - TN10 "I've had to get dermatology approval and I have to go in for blood tests and like monitoring every single month before they prescribe it because it's a very strict drug and very hard to get on." – TN13 "Females who take Accutane have to go to great lengths to make sure there is no pregnancy because Accutane will cause birth defects if you happen to be pregnant while you are taking it." - TN20 “So, every month I had a blood test, I had to do a pregnancy test. And I went up to 30 miligram on month three.” – TN28 "You need to have a blood test, your blood test will be fasting, it will be to check your liver function, your kidney function and your lipids." – TN34 "I like to keep patients on it until we don't see any new acne lesions for about 2 months." – TN45 "So, I'm still on 30mg daily so during the 3rd and 4th week I had to go get my bloods done to make sure I could start my next month, and everything came fine so that's really good." – TN46
	Evaluation for Isotretinoin Candidacy	“So I was prescribed it in quite an unconventional way, so when I was a teenager, I did have recurring issues with my skin, primarily sort of when I started taking birth control, and it was never cystic or anything like that but it did used to really really bother me, on more of like a psychological level.” - TN1 "That's when I started getting some permanent, permanent scarring from my acne, and you know that's where I got really scared and my dermatologist prescribed me with Accutane for the first time." - TN17 "I struggled with acne for many years... started out with blackheads, then hormonal cystic... it just progressively got worse…I had it on my cheeks, my forehead, my back, and my chest... clusters of cystic acne." – TN27 "First I evaluate the patient for the type of acne they have, Accutane is typically reserved for kind of a last case scenario because of all the side effects...we want to make sure that this is a patient who really needs it, so typically Accutane is given out to patients who have pretty severe nodulocystic acne...patients whose acne has not really responded to anything else, like no other medication has touched their acne and they're continuing to break out. Then Accutane might be a possibility…I always consider what other medications the patients are on, the patient cannot be on other medications that will increase their triglycerides or can potentially damage their liver...I always ask my patients of childbearing potential of their family planning goals. Are you planning to get pregnant soon and if you are, then Accutane is not the right choice for you." – TN39 “And everything I tried beforehand, um, was considered and reviewed and then it was decided for me to go on Roaccutane." – TN42 "I reserve it for patients who have failed other acne treatments, or if I see early signs of scarring or current scarring." – TN45
	Ineffective prior treatments	“So, I would go to the doctor, convey the issues that I was having, and they would put me on all these different kinds of antibiotics, I think lymecycline was one of them, they would give me Differin the topical treatment, and none of these things would ever work.” - TN1 "Acne that severe in nature and isn't responding to other medication. Your dermatologist might consider this drug. It's isotretinoin." - TN10 "I've tried absolutely everything else that the doctors can prescribe me and they don't do it, they don't help anything, like nothing's got rid of it like especially long term. I have been using this other spot cream before using these tablets as well so that helps. Nothing's got rid of it like especially long term…But again, once I stop, they come back."- TN13 "I was battling with acne for about 3 years up to that point, and after many treatments from my dermatologist, and I've changed my diet, I've tried literally everything throughout those 3 years nothing was working, antibiotics, everything, topical treatments, creams, nothing, nothing, nothing was working." - TN17 "I started going to a dermatologist... tried everything under the sun... some of it helped a little bit, but not a lot." – TN27 “I'll make another video of the things I tried that did work for a while, but nothing really kind of did it for me” – TN29 "You can do all of the facial cleaning, You can do all of the hydra facials, and you can treat with all of the different antibiotics, but sometimes and in cases like this, Accutane is needed." – TN40 "I tried adapalene, I tried um, try um tretinoin, I tried erythromycin, I tried lymecycline, I tried honestly so much, as well as, like, my own skincare routines." – TN42 "I've been on four or five types of antibiotics, topicals, I've tried herbal remedies, I've tried diet change." – TN43 "I tried different things, like so many different things, different medications, like naturopath, etcetera, gut health blah blah blah, tried all that... I've been on so many medications, tried the holistic route, didn't work for me." – TN44 "I reserve it for patients who have failed other acne treatments." – TN45
	Need for Dermatologist involvement	"There are side effects which you must consider with your dermatologist." – TN24 "If other questions arise, don't hesitate to reach out to your dermatologist!" – TN25 "They are going to discuss the risks and benefits of this medication." - TN26 “Now before I went on it, I had an appointment with a dermatologist um and she basically told me I had to be on contraception for 4 weeks before I could start” – TN28 “Like I mentioned...there is a whole slew of things that dermatologists think about and consider before we would even bring up Accutane to a patient. I rarely even offer Accutane at a first visits unless the patient really needs it. Typically, I like to get to know the patient a bit better and their history a better before even offering it. So, this is a decision that each person makes individually with their dermatologist.” – TN39 "Follow your dermatologists' instructions; they're there to see that journey through with you." – TN41 "I'm a dermatologist, and I prescribe accutane every single day." – TN45
Awareness, Misconceptions, and Communication	Clarifying misconceptions	"Your skin might start to get worse before it starts to improve, especially at the beginning." -TN10 "Accutane is isotretinoin and this TikTok is how isotretinoin may have brought down the size of this guy's nose, or even made it more narrow...This plastic surgery journal talks about using isotretinoin post-operatively after rhinoplasty on those with mestizo noses which is thicker skin, a wider nose, more sebaceous gland heavy… isotretinoin does not replace a rhinoplasty, it did not affect the final cosmetic result one year after surgery." – TN12 “Can you have Botox when you're taking isotretinoin tablets (Roaccutane) for acne? … So, there is no reason why you can inject anti-wrinkle injections into the skin as long as the skin in which you're injecting is not inflamed.” – TN14 "There's a lot of people talking about how Accutane shrinks their nose, or gives them a free nose job... their nose can appear smaller because there is less oil gland tissue...when people talk about their nose looking smaller after they've been on Accutane, it's because they probably had a lot of active oil glands in their nose... Please don't go on Accutane for the sole purpose of shrinking your nose." - TN23 "Now, it does have its side effects, and most are quite quite manageable, but because of that it gets a bad rep online, and often patients dismiss it based on what they've heard from friends or online.” – TN45
	Lack of Information and awareness on Isotretinoin	"I really like this video because I feel like there's not a lot of videos that show the progression of Accutane. It's usually a lot of negativity about it...Let's see the progress, it's doing good." - TN21 "Why the heck are we not talking about this... there are so many popular videos right now that are not giving disclaimers about this type of stuff." – TN22
	Importance of being informed	"Please do your research on these side effects... look up why these side effects happen, you will be shocked…Please remember to do your research and to just be careful what you put into your body." – TN22 "I wouldn't say they love to give it out and they were, they made me very aware of it, not all of them, because as time goes on, I've learned more about the drug itself." – TN42 "I encourage you to talk to a board certified dermatologist if you think you might be a candidate and talk about the risks and benefits because it can totally, totally be worth it." – TN45
	Need for disclaimers	"To all the people that are posting about this medication please remember to add disclaimers... you have no idea if the person that you're recommending this medication to would be able to handle any of these side effects or long-term chronic health issues." – TN22 "It was made known to me that it's a very serious medication and its not to be taken lightly." – TN42
Post-Treatment Considerations and Future Outlook	Reduced severity of acne post-Isotretinoin	"Accutane is generally best for acne which is nodular cystic, and therefore you have a good chance of the rebound not recurring." – TN11 “They did let me know obviously when you finish Accutane early that you're more like, your acne could like come back and stuff like that. I've now been off Accutane for 7 months, and I have been very lucky, I've been very lucky that basically my acne hasn't kind of made a reappearance.” – TN28 "And then I started to see it just clear, and now I would get like, random breakouts, like a couple pimples, and the scarring is the main thing that I have as an issue now." – TN44 "This is my skin, and I couldn't not be happier, I'm just praying it doesn't come back.” -TN47
	Limitations of Accutane in regulating hormones	"Will hormonal acne return after taking Accutane? In short, there's a good chance that it may return because Accutane does not regulate hormones in the way that other drugs do." – TN11 “What we have found is that hormonal acne does tend to get better with Accutane … but for patients with hormonal acne, once they stop the Accutane their acne can come back. So, Accutane doesn't have the same type of lasting effect on their acne as it does for other patients with run of the mill cystic acne.” – TN39
	Post-Accutane health struggles	"There are so many people that are struggling with their health and their skin post Accutane, there really needs to be more awareness about the downside of Accutane... a lot of people are really suffering." – TN22
	Post-Accutane Treatment Considerations	"It may not be as severe as it was before taking Accutane, but sometimes it's worthwhile considering using either topical or oral spironolactone." – TN11 "I started tackling it in other ways, trying to find skincare routines that will really work for my face, and will help it get to the point where I want it in a more natural and different topical way. So this is how I started the ordinary and if you want to see results and the journey, please follow me so you can check it out." - TN17 “This means that my patients with hormonal acne still need something to treat their acne even after a round of Accutane. What could that something be depends on how badly their hormonal acne recurs. Options for hormonal acne depending on your risk factors include oral spironolactone, topical spironolactone ... or possibly birth control pills.” – TN39 “Basically, while I was on Roaccutane, I didn't get my period, so I got my period for the first time 4 weeks ago, and my skin broke out like hell on my period and I was like oh no, so I switched up my skin care." – TN47
